# Optimal convex combination bounds of geometric and Neuman means for Toader-type mean

**DOI:** 10.1186/s13660-017-1473-1

**Published:** 2017-08-29

**Authors:** Yue-Ying Yang, Wei-Mao Qian

**Affiliations:** 1 School of Mechanical and Electrical Engineering, Huzhou Vocational & Technical College, Huzhou, 313000 China; 2 School of Distance Education, Huzhou Broadcast and TV University, Huzhou, 313000 China

**Keywords:** 26E60, 33E05, Toader mean, geometric mean, Neuman mean

## Abstract

In this paper, we prove that the double inequalities

$$\begin{aligned}& {\alpha }N_{QA}(a,b)+({1-\alpha })G(a,b)< TD \bigl[A(a,b),G(a,b) \bigr]< {\beta }N_{QA}(a,b)+({1-\beta })G(a,b), \\& {\lambda }N_{AQ}(a,b)+({1-\lambda })G(a,b)< TD \bigl[A(a,b),G(a,b) \bigr]< {\mu }N_{AQ}(a,b)+({1-\mu })G(a,b) \end{aligned}$$

hold for all $a,b>0$ with $a\neq b$ if and only if $\alpha \leq 3/8$, $\beta \geq 4/ [\pi ( \log (1+\sqrt{2})+\sqrt{2}) ]=0.5546 \cdots $ , $\lambda \leq 3/10$ and $\mu \geq 8/ [\pi (\pi +2) ]=0.4952 \cdots $ , where $TD(a,b)$, $G(a,b)$, $A(a,b)$ and $N_{QA}(a,b)$, $N_{AQ}(a,b)$ are the Toader, geometric, arithmetic and two Neuman means of *a* and *b*, respectively.

## Introduction

For $x,y,z \geq 0$ with $xy+xz+yz\neq 0$ and $r\in (0,1)$, the symmetric integrals $R_{F}(x,y,z)$ and $R_{G}(x,y,z)$ [1] of the first and second kinds, and the complete elliptic integrals $\mathcal{K}(r)$ and $\mathcal{E}(r)$ of the first and second kinds are defined by

$$\begin{aligned}& R_{F}(x,y,z) =\frac{1}{2} \int_{0}^{\infty } \bigl[(t+x) (t+y) (t+z) \bigr]^{-1/2}\,dt, \\& R_{G}(x,y,z) =\frac{1}{4} \int_{0}^{\infty } \bigl[(t+x) (t+y) (t+z) \bigr]^{-1/2} \biggl(\frac{x}{t+x}+\frac{y}{t+y}+\frac{z}{t+z} \biggr)t\,dt, \\& \mathcal{K}(r)= \int_{0}^{\pi /2} \bigl[1-r^{2} \sin^{2}(t) \bigr]^{-1/2}\,dt, \qquad \mathcal{E}(r)= \int_{0}^{\pi /2} \bigl[1-r^{2} \sin^{2}(t) \bigr]^{1/2}\,dt, \end{aligned}$$

respectively.

The well-known identities

$$\mathcal{K}(r)=R_{F}\bigl(0,1-r^{2},1\bigr),\qquad \mathcal{E}(r)=2 R_{G}\bigl(0,1-r^{2},1\bigr) $$

were established by Carlson in [1].

Let $a,b>0$ with $a\neq b$. Then the Toader mean $\mathit{TD}(a,b)$ [2] and the Schwab-Borchardt mean $SB(a,b)$ [3–5] are respectively defined by

1.1 $$\begin{aligned} TD(a,b) =& \frac{2}{\pi } \int_{0}^{\pi /2}\sqrt{a^{2} \cos^{2}(t)+b ^{2}\sin^{2}(t)}\,dt \\ =& \textstyle\begin{cases} 2a\mathcal{E} (\sqrt{1-(b/a)^{2}} )/\pi, & a>b, \cr 2b\mathcal{E} (\sqrt{1-(a/b)^{2}} )/\pi, & a< b, \end{cases}\displaystyle \end{aligned}$$

and

$$SB(a,b)= \textstyle\begin{cases} \frac{\sqrt{b^{2}-a^{2}}}{\cos^{-1}(a/b)}, & a< b, \\ \frac{\sqrt{a^{2}-b^{2}}}{\cosh^{-1}(a/b)}, & a>b, \end{cases} $$

where $\cos^{-1}(x)$ and $\cosh^{-1}(x)=\log (x+\sqrt{x^{2}-1})$ are the inverse cosine and inverse hyperbolic cosine functions, respectively.

Very recently, Neuman [6] introduced the Neuman mean $N(a,b)$ of the second kind as follows:

$$N(a,b)=\frac{1}{2} \biggl[a+\frac{b^{2}}{SB(a,b)} \biggr]. $$

It is well known that the Toader mean $TD(a,b)$, the Schwab-Borchardt mean $SB(a,b)$ and the Neuman mean of the second kind $N(a,b)$ satisfy the identities (see [6, 7])

$$\begin{aligned}& \begin{aligned} TD(a,b) &=\frac{4}{\pi }R_{G}\bigl(a^{2},b^{2},0 \bigr) \\ &=\frac{1}{\pi } \int_{0} ^{\infty } \bigl[\bigl(t+a^{2}\bigr) \bigl(t+b^{2}\bigr) \bigr]^{-1/2} \biggl(\frac{a^{2}}{t+a ^{2}}+ \frac{b^{2}}{t+b^{2}} \biggr)t\,dt, \end{aligned} \\& \begin{aligned} SB(a,b) &=1/R_{F}\bigl(a^{2},b^{2},b^{2} \bigr) \\ &=2/ \int_{0}^{\infty } \bigl[\bigl(t+a^{2}\bigr) \bigl(t+b ^{2}\bigr) \bigl(t+b^{2}\bigr) \bigr]^{-1/2}\,dt, \end{aligned} \\& \begin{aligned} N(a,b) &=R_{G}\bigl(a^{2},b^{2},b^{2} \bigr) \\ &=\frac{1}{4} \int_{0}^{\infty } \bigl[\bigl(t+a^{2}\bigr) \bigl(t+b^{2}\bigr) \bigl(t+b^{2}\bigr) \bigr]^{-1/2} \biggl(\frac{a^{2}}{t+a^{2}}+\frac{b^{2}}{t+b^{2}}+ \frac{b^{2}}{t+b^{2}} \biggr)t\,dt. \end{aligned} \end{aligned}$$

Let $p\in \mathbb{R}$ and $a,b>0$. Then the *p*th power mean $M_{p}(a,b)$ is defined by

1.2 $$ M_{p}(a,b) = \bigl[\bigl(a^{p}+b^{p} \bigr)/2 \bigr]^{1/p}(p\neq 0),\qquad M_{0}(a,b)= \sqrt{ab}. $$

We clearly see that $M_{p}(a,b)$ is symmetric and homogeneous of degree one with respect to *a* and *b*, strictly increasing with respect to $p\in \mathbb{R}$ for fixed $a,b>0$ with $a\neq b$, and the inequalities

$$G(a,b)=M_{0}(a,b)< A(a,b)=M_{1}(a,b)< Q(a,b)=M_{2}(a,b) $$

hold for $a,b>0$ with $a\neq b$, where $G(a,b)=\sqrt{ab}$, $A(a,b)=(a+b)/2$ and $Q(a,b)=\sqrt{(a^{2}+b^{2})/2}$ are the geometric, arithmetic and quadratic means of *a* and *b*, respectively.

In [6], Neuman presented the explicit formula for $N_{QA}(a,b) \equiv N[Q(a,b),A(a,b)]$ and $N_{AQ}(a,b)\equiv N[A(a,b),Q(a,b)]$ as follows:

1.3 $$\begin{aligned}& N_{QA}(a,b)=\frac{1}{2}A(a,b) \biggl[ \sqrt{1+v^{2}}+ \frac{\sinh^{-1}(v)}{v} \biggr], \end{aligned}$$

1.4 $$\begin{aligned}& N_{AQ}(a,b)=\frac{1}{2}A(a,b) \biggl[1+ \bigl(1+v^{2}\bigr)\frac{\tan^{-1}(v)}{v} \biggr] \end{aligned}$$

and proved that the inequalities

1.5 $$ A(a,b)< N_{QA}(a,b)< N_{AQ}(a,b)< Q(a,b) $$

hold for $a,b>0$ with $a\neq b$, where $v=(a-b)/(a+b)$.

Recently, the Toader mean has been the subject of intensive research. In particular, many remarkable inequalities for Toader mean and other related means can be found in the literature [8–41].

In [42], Vuorinen conjectured that

$$TD(a,b)>M_{3/2}(a,b) $$

for all $a,b>0$ with $a\neq b$. This conjecture was proved by Qiu and Shen [43], and Barnard et al. [44], respectively, and Alzer and Qiu [45] presented the best possible upper power mean bound for the Toader mean as follows:

$$TD(a,b)< M_{\log 2/\log (\pi /2)}(a,b) $$

for all $a,b>0$ with $a\neq b$.

Li, Qian and Chu [46] proved that the inequality

$$\alpha N_{AQ}(a,b)+(1-\alpha)A(a,b)< TD(a,b)< \beta N_{AQ}(a,b)+(1- \beta)A(a,b) $$

holds for all $a,b>0$ with $a\neq b$ if and only if $\alpha \leq 3/4$ and $\beta \geq 4(4-\pi)/ [\pi (\pi -2) ]=0.9573\cdots $ .

Note that

1.6 $$ G(a,b)< TD \bigl[A(a,b),G(a,b) \bigr]< A(a,b) $$

for all $a,b>0$ with $a\neq b$.

From inequalities (1.5) and (1.6) we clearly see that

$$G(a,b)< TD \bigl[A(a,b),G(a,b) \bigr]< N_{QA}(a,b)< N_{AQ}(a,b) $$

for all $a,b>0$ with $a\neq b$.

The main purpose of this paper is to find the greatest values *α*, *λ* and the least values *β*, *μ* such that the double inequalities

$$\begin{aligned}& \alpha N_{QA}(a,b)+(1-\alpha)G(a,b)< TD \bigl[A(a,b),G(a,b) \bigr]< \beta N_{QA}(a,b)+(1-\beta)G(a,b), \\& \lambda N_{AQ}(a,b)+(1-\lambda)G(a,b)< TD \bigl[A(a,b),G(a,b) \bigr]< \mu N_{AQ}(a,b)+(1-\mu)G(a,b) \end{aligned}$$

hold for all $a,b>0$ with $a\neq b$. As applications, we get two new bounds for the complete elliptic integral of the second kind in terms of elementary functions.

## Lemmas

In order to prove our main results, we need several lemmas, which we present in this section.

For $r\in (0,1)$, we clearly see that

$$\mathcal{K}\bigl(0^{+}\bigr)=\mathcal{E}\bigl(0^{+}\bigr)= \pi /2, \qquad \mathcal{K}\bigl(1^{-}\bigr)=+ \infty,\qquad \mathcal{E} \bigl(1^{-}\bigr)=1, $$

and $\mathcal{K}(r)$ and $\mathcal{E}(r)$ satisfy the formulas (see[21], Appendix E, pp.474-475)

$$\begin{aligned}& \frac{d\mathcal{K}(r)}{dr}=\frac{\mathcal{E}(r)-(1-r^{2})\mathcal{K}(r)}{r(1-r ^{2})}, \qquad \frac{d\mathcal{E}(r)}{dr}= \frac{\mathcal{E}(r)-\mathcal{K}(r)}{r}, \\& \frac{d [\mathcal{E}(r)-\mathcal{K}(r) ]}{dr}=-\frac{r \mathcal{E}(r)}{1-r^{2}}. \end{aligned}$$

### Lemma 2.1

see [21], Theorem 1.25

*For* $-\infty < a< b<+\infty $, *let* $f,g:[a,b]\rightarrow \mathbb{R}$ *be continuous on* $[a,b]$ *and differentiable on* $(a,b)$, *and* $g'(x)\neq 0$ *on* $(a,b)$. *If* $f'(x)/g'(x)$ *is increasing* (*decreasing*) *on* $(a,b)$, *then so are*

$$\frac{f(x)-f(a)}{g(x)-g(a)}\quad \textit{and}\quad \frac{f(x)-f(b)}{g(x)-g(b)}. $$

*If* $f'(x)/g'(x)$ *is strictly monotone*, *then the monotonicity in the conclusion is also strict*.

### Lemma 2.2

see [21], Theorem 3.21(1), Exercise 3.43(11) and Exercise 3.43(29)

1. *The function* $r\mapsto [\mathcal{E}(r)-(1-r^{2})\mathcal{K}(r) ]/r^{2}$ *is strictly increasing from* $(0,1)$ *onto* $(\pi /4,1)$;
2. *The function* $r\mapsto [\mathcal{K}(r)-\mathcal{E}(r) ]/r ^{2}$ *is strictly increasing from* $(0,1)$ *onto* $(\pi /4,+\infty)$;
3. *The function* $r\mapsto [(2-r^{2})\mathcal{K}(r)-2\mathcal{E}(r) ]/r^{4}$ *is strictly increasing from* $(0,1)$ *onto* $(\pi /16,+ \infty)$.

### Lemma 2.3

*The function* $r\mapsto \varphi_{1}(r)= \{\frac{2}{\pi }\sqrt{1-r ^{2}} [2\mathcal{E}(r)-\mathcal{K}(r) ]+2r^{2}-1 \}/r^{2}$ *is strictly increasing from* $(0,1)$ *onto* $(3/4,1)$.

### Proof

Simple computations lead to

2.1 $$\begin{aligned}& \varphi_{1}\bigl(0^{+}\bigr)= \frac{3}{4},\qquad \varphi_{1}\bigl(1^{-}\bigr)=1, \end{aligned}$$

2.2 $$\begin{aligned}& \varphi_{1}'(r)=\frac{2}{\pi r^{3}} \gamma_{1}(r), \end{aligned}$$

where

2.3 $$\begin{aligned}& \gamma_{1}(r)=\frac{\mathcal{K}(r)-3\mathcal{E}(r)}{\sqrt{1-r^{2}}}+ \pi, \end{aligned}$$

2.4 $$\begin{aligned}& \gamma_{1}\bigl(0^{+}\bigr)=0, \end{aligned}$$

2.5 $$\begin{aligned}& \gamma_{1}'(r)=\frac{r^{3}}{(1-r^{2})^{3/2}} \frac{(2-r^{2}) \mathcal{K}(r)-2\mathcal{E}(r)}{r^{4}}. \end{aligned}$$

From (2.5) and Lemma 2.2(3) we get

2.6 $$ \gamma_{1}'(r)>\frac{\pi r^{3}}{16(1-r^{2})^{3/2}}>0. $$

Therefore, Lemma 2.3 follows easily from (2.1), (2.2), (2.4) and (2.6). □

### Lemma 2.4

*The function* $r\mapsto \varphi_{2}(r)=(2r^{2}+\sqrt{1-r^{4}}-1)/r ^{2}$ *is strictly decreasing from* $(0,1)$ *onto* $(1,2)$.

### Proof

It is easy to verify that

2.7 $$\begin{aligned}& \varphi_{2}\bigl(0^{+}\bigr)=2,\qquad \varphi_{2}\bigl(1^{-}\bigr)=1, \end{aligned}$$

2.8 $$\begin{aligned}& \varphi_{2}'(r)=\frac{2(\sqrt{1-r^{4}}-1)}{r^{3} \sqrt{1-r^{4}}}< 0 \end{aligned}$$

for $r\in (0,1)$.

Therefore, Lemma 2.4 follows easily from (2.7) and (2.8). □

### Lemma 2.5

*The function* $r\mapsto \varphi_{3}(r)= [2r^{2}\mathcal{K}(r)-5 \mathcal{E}(r) ]/\sqrt{1-r^{2}}$ *is strictly increasing from* $(0,1)$ *onto* $(-5\pi /2,+\infty)$.

### Proof

It is not difficult to verify that

2.9 $$\begin{aligned}& \varphi_{3}\bigl(0^{+}\bigr)=- \frac{5}{2}\pi,\qquad \varphi_{3}\bigl(1^{-}\bigr)=+\infty, \end{aligned}$$

2.10 $$\begin{aligned}& \varphi_{3}'(r)=\frac{r}{(1-r^{2})^{3/2}} \biggl[\bigl(5-3r^{2}\bigr)\frac{ \mathcal{K}(r)-\mathcal{E}(r)}{r^{2}}-\mathcal{E}(r) \biggr]. \end{aligned}$$

From (2.10) and Lemma 2.2(2) together with the monotonicity of $\mathcal{E}(r)$ on $(0,1)$ we clearly see that

2.11 $$ \varphi_{3}'(r)>\frac{r}{(1-r^{2})^{3/2}} \biggl[\bigl(5-3r^{2}\bigr)\times \frac{ \pi }{4}-\frac{\pi }{2} \biggr]=\frac{3\pi }{4} \frac{r}{\sqrt{1-r^{2}}}>0 $$

for $r\in (0,1)$.

Therefore, Lemma 2.5 follows from (2.9) and (2.11). □

### Lemma 2.6

*The function* $r\mapsto \varphi_{4}(r)= \{\frac{2}{\pi }\sqrt{1-r ^{2}} [2\mathcal{E}(r)-(1+r^{2})\mathcal{K}(r) ]+3r^{2}-1 \}/r^{2}$ *is strictly increasing from* $(0,1)$ *onto* $(3/4,2)$.

### Proof

Let $\phi_{1}(r)=\frac{2}{\pi }\sqrt{1-r^{2}} [2\mathcal{E}(r)-(1+r ^{2})\mathcal{K}(r) ]+3r^{2}-1$, $\phi_{2}(r)=r^{2}$. Then simple computations give

2.12 $$\begin{aligned}& \phi_{1}\bigl(0^{+}\bigr)= \phi_{2}(0)=0,\qquad \varphi_{4}(r)=\phi_{1}(r)/\phi _{2}(r), \end{aligned}$$

2.13 $$\begin{aligned}& \varphi_{4}\bigl(1^{-}\bigr)=2, \end{aligned}$$

2.14 $$\begin{aligned}& \frac{\phi_{1}'(r)}{\phi_{2}'(r)}=3+\frac{1}{\pi \sqrt{1-r^{2}}} \biggl[\frac{\mathcal{E}(r)-(1-r^{2})\mathcal{K}(r)}{r^{2}} \biggr]+\frac{1}{ \pi }\varphi_{3}(r). \end{aligned}$$

It follows from Lemma 2.2(1), Lemma 2.5 and the function $r\mapsto \sqrt{1-r^{2}}$ strictly decreasing that $\phi_{1}'(r)/ \phi_{2}'(r)$ is strictly increasing on $(0,1)$ and

2.15 $$ \varphi_{4}\bigl(0^{+}\bigr)=\lim _{r\rightarrow 0^{+}}\frac{\phi_{1}'(r)}{ \phi_{2}'(r)}=\frac{3}{4}. $$

Therefore, Lemma 2.6 follows from Lemma 2.1, (2.12), (2.13) and (2.15) together with the monotonicity of $\phi_{1}'(r)/\phi_{2}'(r)$. □

### Lemma 2.7

*The function* $\varphi_{5}(r)= [3r^{2} +\sqrt{1-r^{2}}-1 ]/r ^{2}$ *is strictly decreasing from* $(0,1)$ *onto* $(2,5/2)$.

### Proof

We clearly see that

2.16 $$\begin{aligned}& \varphi_{5}\bigl(0^{+}\bigr)= \frac{5}{2},\qquad \varphi_{5}\bigl(1^{-}\bigr)=2, \end{aligned}$$

2.17 $$\begin{aligned}& \varphi_{5}'(r)=-\frac{(1-\sqrt{1-r^{2}})^{2}}{r^{3} \sqrt{1-r ^{2}}}< 0 \end{aligned}$$

for $r\in (0,1)$.

Therefore, Lemma 2.7 follows easily from (2.16) and (2.17). □

## Main results

### Theorem 3.1

*The double inequality*

3.1 $$ \alpha N_{QA}(a,b)+(1-\alpha)G(a,b)< TD \bigl[A(a,b),G(a,b) \bigr]< \beta N_{QA}(a,b)+(1-\beta)G(a,b) $$

*holds for all* $a,b>0$ *with* $a\neq b$ *if and only if* $\alpha \leq 3/8$ *and* $\beta \geq 4/ [\pi (\log (1+\sqrt{2})+\sqrt{2} ) ]=0.5546\cdots $ .

### Proof

Since $G(a,b)$, $TD(a,b)$ and $N_{QA}(a,b)$ are symmetric and homogenous of degree 1, without loss of generality, we assume that $a>b>0$ and let $r=(a-b)/(a+b)\in (0,1)$. Then (1.1)-(1.3) lead to

3.2 $$\begin{aligned}& TD \bigl[A(a,b),G(a,b) \bigr]=\frac{2}{\pi }A(a,b) \mathcal{E}(r), \end{aligned}$$

3.3 $$\begin{aligned}& G(a,b)=A(a,b)\sqrt{1-r^{2}},\qquad N_{QA}(a,b)= \frac{1}{2}A(a,b) \biggl[\sqrt{1+r ^{2}}+\frac{\sinh^{-1}(r)}{r} \biggr]. \end{aligned}$$

It follows from (3.2)-(3.3) that

3.4 $$\begin{aligned}& \frac{T [A(a,b),G(a,b) ]-G(a,b)}{N_{QA}(a,b)-G(a,b)} \\& \quad =\frac{\frac{2}{ \pi }\varepsilon (r)-\sqrt{1-r^{2}}}{\frac{1}{2} [\sqrt{1+r ^{2}}+\frac{\sinh^{-1}(r)}{r} ]-\sqrt{1-r^{2}}} \\& \quad =\frac{\frac{4}{\pi }r\varepsilon (r)-2r \sqrt{1-r^{2}}}{\sinh^{-1}(r)+ (r\sqrt{1+r^{2}}-2r \sqrt{1-r^{2}} )}. \end{aligned}$$

Let $f_{1}(r)=\frac{4}{\pi }r\varepsilon (r)-2r \sqrt{1-r^{2}}$, $f_{2}(r)=\sinh^{-1}(r)+ (r\sqrt{1+r^{2}}-2r \sqrt{1-r^{2}} )$ and

3.5 $$ f(r)=\frac{\frac{4}{\pi }r\varepsilon (r)-2r \sqrt{1-r^{2}}}{\sinh ^{-1}(r)+ (r\sqrt{1+r^{2}}-2r \sqrt{1-r^{2}} )}. $$

Then simple computations lead to

3.6 $$\begin{aligned}& f_{1}\bigl(0^{+}\bigr)=f_{2}(0)=0, \end{aligned}$$

3.7 $$\begin{aligned}& \frac{f_{1}'(r)}{f_{2}'(r)}=\frac{\frac{2}{\pi }\sqrt{1-r^{2}} [2\varepsilon (r)-\kappa (r) ]+2r^{2}-1 }{2r^{2}+\sqrt{1-r ^{4}}-1}=\frac{\varphi_{1}(r)}{\varphi_{2}(r)}, \end{aligned}$$

where $\varphi_{1}(r)$ and $\varphi_{2}(r)$ are defined as in Lemmas 2.3 and 2.4.

It follows from Lemmas 2.3-2.4 and (3.7) that $f_{1}'(r)/f_{2}'(r)$ is strictly increasing on $(0,1)$. Then (3.5), (3.6) and Lemma 2.1 lead to the conclusion that $f(r)$ is strictly increasing.

Moreover,

3.8 $$\begin{aligned}& \lim_{r\rightarrow 0^{+}}\frac{\frac{4}{\pi }r\varepsilon (r)-2r \sqrt{1-r^{2}}}{\sinh^{-1}(r)+ (r\sqrt{1+r^{2}}-2r \sqrt{1-r ^{2}} )}= \frac{3}{8}, \end{aligned}$$

3.9 $$\begin{aligned}& \lim_{r\rightarrow 1^{-}}\frac{\frac{4}{\pi }r\varepsilon (r)-2r \sqrt{1-r^{2}}}{\sinh^{-1}(r)+ (r\sqrt{1+r^{2}}-2r \sqrt{1-r ^{2}} )}= \frac{4}{\pi [\pi (\log (1+\sqrt{2})+\sqrt{2} ) ]}. \end{aligned}$$

Therefore, Theorem 3.1 follows easily from (3.4), (3.8) and (3.9) together with the monotonicity of $f(r)$. □

### Theorem 3.2

*The double inequality*

3.10 $$\begin{aligned} \lambda N_{AQ}(a,b)+(1-\lambda)G(a,b) < &TD \bigl[A(a,b),G(a,b) \bigr] \\ < & \mu N_{AQ}(a,b)+(1-\mu)G(a,b) \end{aligned}$$

*holds for all* $a,b>0$ *with* $a\neq b$ *if and only if* $\lambda \leq 3/10$ *and* $\mu \geq 8/ [\pi (\pi +2) ]=0.4952\cdots $ .

### Proof

Without loss of generality, we assume that $a>b>0$ and let $r=(a-b)/(a+b) \in (0,1)$. Then from (1.4) we get

3.11 $$ N_{AQ}(a,b)=\frac{1}{2}A(a,b) \biggl[1+ \bigl(1+r^{2}\bigr) \frac{\tan^{-1}(r)}{r} \biggr]. $$

It follows from (3.2), (3.11) and $G(a,b)=A(a,b)\sqrt{1-r ^{2}}$ that

3.12 $$\begin{aligned}& \frac{TD [A(a,b),G(a,b) ]-G(a,b)}{N_{AQ}(a,b)-G(a,b)} \frac{\frac{2}{ \pi }\mathcal{E}(r)-\sqrt{1-r^{2}}}{\frac{1}{2} [1+(1+r^{2})\frac{ \tan^{-1}(r)}{r} ]-\sqrt{1-r^{2}}} \\& \quad =\frac{ [\frac{4}{\pi }r\mathcal{E}(r)-2r \sqrt{1-r^{2}} ]/(1+r ^{2})}{\tan^{-1}(r)+ (r-2r \sqrt{1-r^{2}} )/(1+r^{2})}. \end{aligned}$$

Let $g_{1}(r)= [\frac{4}{\pi }r\mathcal{E}(r)-2r \sqrt{1-r^{2}} ]/(1+r^{2})$, $g_{2}(r)=\tan^{-1}(r)+ (r-2r \sqrt{1-r^{2}} ) /(1+r^{2})$ and

3.13 $$ g(r)=\frac{ [\frac{4}{\pi }r\mathcal{E}(r)-2r\sqrt{1-r^{2}} ]/(1+r^{2})}{\tan^{-1}(r)+ (r-2r \sqrt{1-r^{2}} )/(1+r ^{2})}. $$

Then simple computations lead to

3.14 $$\begin{aligned}& g_{1}\bigl(0^{+}\bigr)=g_{2}(0)=0, \end{aligned}$$

3.15 $$\begin{aligned}& \frac{g_{1}'(r)}{g_{2}'(r)}=\frac{\frac{2}{\pi }\sqrt{1-r^{2}} [2\varepsilon (r)-(1+r^{2})\kappa (r) ]+3r^{2}-1 }{3r^{2}+\sqrt{1-r ^{2}}-1}=\frac{\varphi_{4}(r)}{\varphi_{5}(r)}, \end{aligned}$$

where $\varphi_{4}(r)$ and $\varphi_{5}(r)$ are defined as in Lemmas 2.6 and 2.7.

It follows from Lemmas 2.6-2.7 and (3.15) that $g_{1}'(r)/g_{2}'(r)$ is strictly increasing on $(0,1)$. Then (3.13), (3.14) and Lemma 2.1 lead to the conclusion that $g(r)$ is strictly increasing.

Moreover,

3.16 $$\begin{aligned}& \lim_{r\rightarrow 0^{+}}\frac{ [\frac{4}{\pi }r\varepsilon (r)-2r \sqrt{1-r^{2}} ]/(1+r^{2})}{\tan^{-1}(r)+ (r-2r \sqrt{1-r ^{2}} )/(1+r^{2})}= \frac{3}{10}, \end{aligned}$$

3.17 $$\begin{aligned}& \lim_{r\rightarrow 1^{-}}\frac{ [\frac{4}{\pi }r\varepsilon (r)-2r \sqrt{1-r^{2}} ]/(1+r^{2})}{\tan^{-1}(r)+ (r-2r \sqrt{1-r ^{2}} )/(1+r^{2})}= \frac{8}{\pi (\pi +2)}. \end{aligned}$$

Therefore, Theorem 3.2 follows from (3.12), (3.16) and (3.17) together with the monotonicity of $g(r)$. □

From Theorems 3.1-3.2 we get the following Corollary 3.3 immediately.

### Corollary 3.3

*Let* $\alpha =3/8$, $\beta =4/ [\pi (\log (1+\sqrt{2})+ \sqrt{2} ) ]=0.5546\cdots $ , $\lambda =3/10$ *and* $\mu =8/ [ \pi (\pi +2) ]=0.4952\cdots $ . *Then the double inequalities*

$$\begin{aligned}& \frac{1}{4}\pi \alpha \biggl[\sqrt{1+r^{2}}+\frac{\sinh^{-1}(r)}{r} \biggr]+\frac{1}{2}\pi (1- \alpha)\sqrt{1-r^{2}} \\& \quad < \mathcal{E}(r)< \frac{1}{4}\pi \beta \biggl[\sqrt{1+r^{2}}+\frac{\sinh^{-1}(r)}{r} \biggr]+\frac{1}{2}\pi (1- \beta)\sqrt{1-r^{2}}, \\& \frac{1}{4}\pi \lambda \biggl[1+\bigl(1+r^{2}\bigr) \frac{\tan^{-1}(r)}{r} \biggr]+ \frac{1}{2}\pi (1- \lambda) \sqrt{1-r^{2}} \\& \quad < \mathcal{E}(r)< \frac{1}{4}\pi \mu \biggl[1+\bigl(1+r^{2}\bigr) \frac{\tan^{-1}(r)}{r} \biggr]+ \frac{1}{2}\pi (1- \mu)\sqrt{1-r^{2}} \end{aligned}$$

*hold for all* $r\in (0,1)$.

## Results and discussion

In this paper, we provide the sharp bounds for the Toader-type mean in terms of the convex combination of geometric and Neuman means. As applications, we find new bounds for the complete elliptic integral of the second kind.

## Conclusion

In the article, we present the optimal convex combination bounds of the geometric and Neuman means for the Toader-type mean, and give several new upper and lower bounds for the complete elliptic integral of the second kind. The given results are the improvements of some previously known results.
